# Enhanced Attentional Bias Variability in Post-Traumatic Stress Disorder and its Relationship to More General Impairments in Cognitive Control

**DOI:** 10.1038/s41598-017-15226-7

**Published:** 2017-11-06

**Authors:** Diane Swick, Victoria Ashley

**Affiliations:** 1 VA Northern California Health Care System, Martinez, CA USA; 2 University of California, Davis, USA

## Abstract

Hypervigilance towards threat is one of the defining features of post-traumatic stress disorder (PTSD). This symptom predicts that individuals with PTSD will be biased to attend to potential dangers in the environment. However, cognitive tasks designed to assess visual-spatial attentional biases have shown mixed results. A newer proposal suggests that attentional bias is not a static phenomenon, but rather is characterized by fluctuations towards and away from threat. Here, we tested 28 combat Veterans with PTSD and 28 control Veterans on a dot probe task with negative-neutral word pairs. Combat-related words and generically negative words were presented in separate blocks. Replicating previous results, neither group showed a bias to attend towards or away from threat, but PTSD patients showed greater attentional bias variability (ABV), which correlated with symptom severity. However, the cognitive processes indexed by ABV are unclear. The present results indicated that ABV was strongly correlated with standard deviation at the reaction time (RT) level and with excessively long RTs (ex-Gaussian tau) related to cognitive failures. These findings suggest an overall increase in response variability unrelated to threat-related biases in spatial attention, and support a disruption in more general cognitive control processes in PTSD.

## Introduction

Post-traumatic stress disorder (PTSD) is a disabling psychological condition that can develop after exposure to traumatic events. An exaggerated fear response and dysregulation of prefrontal-amygdala circuits are core features of PTSD^1^. Hyperarousal is a key symptom cluster (DSM-5, American Psychiatric Association, 2013)^2^, characterized by hypervigilance to surroundings, increased startle, and insomnia. This clinical feature leads to the prediction that PTSD patients are overly attentive to threats (or potential threats) in the environment.

Individuals with PTSD do show a bias to attend towards trauma-relevant stimuli in some cognitive studies^3–7^ This bias can manifest as facilitation in performance (improved orienting or target detection) or as interference (delayed disengagement from the stimulus). However, other experiments have found a bias *away* from threatening stimuli^8–10^ or no bias at all^11^. A number of factors contribute to these discrepancies in the literature. One issue is that attention bias has been assessed using different tasks. Two of the major tasks are the emotional Stroop and the dot-probe task. These tasks are considered measures of threat-related interference and visual-spatial attention bias, respectively^12^.

In the emotional Stroop task, participants name the ink color of printed words that are negative (e.g., trauma-related) or neutral in valence while ignoring the word^13^. Slowing of response times (RTs) for naming the color of negative words, relative to neutral words, indexes the emotional Stroop interference effect. Many studies have shown that participants with PTSD have a greater emotional Stroop effect than controls^14–18^ but see^8^ for conditions where this is not the case). In our previous study, blocks of 84 unique combat-related words, non-combat negative words, and matched neutral words were presented to combat Veterans with PTSD, Veteran controls, and civilian controls^3^. The emotional Stroop effect for combat words was nearly three times larger in the patient group than in the Veteran controls, who did not differ from the civilian controls. The exaggerated Stroop effect was specific for trauma-related words, and was not seen for non-combat negative words.

In contrast to the emotional Stroop, the literature is mixed on whether PTSD patients show an attentional bias when the dot probe task is used. Some studies show no bias^11,19^ others a bias towards threat^20,6^ and still others a bias *away* from threat^9,10,21,22^. In the dot probe task, two cue stimuli (pictures, faces, or words) of differing valences are presented, followed by a probe stimulus in one of the two cue locations^23^. A bias towards or away from threat is determined by calculating the difference in RT to the emotional and neutral items. When this bias index (neutral – emotional) is positive, attentional bias is directed towards the emotional stimuli. An influential meta-analysis indicated that individuals with anxiety disorders are biased to direct attention towards threatening stimuli^4^. However, recent studies have observed large individual differences in the size and direction of attentional bias in PTSD^24,25^ similar to reports in other anxiety disorders^26^. This widespread variation complicates use of the dot probe task as a standard measure of bias. Furthermore, split-half studies have shown that RT data from the dot probe task do not provide an internally reliable measure of attentional bias to threat^27–29^.

Because of the between-subject variability and within-subject unreliability of the bias index measure, researchers have developed other ways to assess bias. Attention bias variability (ABV) is a relatively new metric that takes advantage of within-subject inconsistencies in bias towards or away from threat over the course of testing^19^. The idea is that individuals with PTSD show a deficit in attentional control when threatening stimuli are present, which leads to greater trial-to-trial variability in attending towards or away from threat. Several studies have reported that ABV is elevated in participants with PTSD^19,30^. This impairment was specific to PTSD, and was not seen in participants with high anxiety scores or social anxiety^30^. However, in the absence of any attentional bias within a testing session (as was the case in those studies), ABV is more likely a reflection of measurement error than bias variability^31^. In fact, simulations using randomly generated data demonstrated that ABV is sensitive to increases in mean RT and increases in standard deviation (SD) at the RT level, but not to increases in bias^31^. Since patient groups often have longer and more variable RTs than controls, some group comparisons of ABV may be misleading. Nonetheless, this type of “measurement error” can be a rich source of data.

Response variability is a highly investigated topic in populations with attention difficulties, most notably attention deficit hyperactivity disorder (ADHD)^32,33^. An increase in trial-to-trial variability of RT in cognitive tasks is seen as a marker of executive dysfunction^34^ and even “mental noise”^35^. In this literature, response variability is assessed by within-subject SD and by the intra-individual coefficient of variation (ICV), which takes into account the speed of responding^36^. Although ICV would not specifically assess fluctuations in attention bias towards and away from threat in the dot probe task, it is not subject to the shortcomings of ABV and another new index of bias dynamics, Trial Level Bias Score (TL-BS) variability^37^.

Our previous work indicated that PTSD patients showed more variable RTs in a Go/NoGo task in the absence of mean RT differences^38^. The effect size was large + (*d* = 0.81), and RT variability was strongly correlated with PTSD symptom severity. Furthermore, response variability predicted whether an individual would have PTSD, even after controlling for symptom severity. These results suggest that response variability in PTSD is not unique to contexts of threat bias, but is also seen in tasks that use neutral stimuli. Thus, it is important to consider that an increase in ABV in the dot probe task may not only reflect fluctuations in attentional bias, but also a more general difficulty in cognitive control.

Assessing the distributional aspects of RT data can be an important addition to the overall picture. Is greater within-subject variability in the patients due to a larger number of slow responses in the rightward tail of the RT distribution, and/or to greater spread around the mean? RT distributions can be characterized by two components: the Gaussian (normal) distribution and an exponential component, the rightward skew or tail consisting of long RTs^39,40^. The ex-Gaussian model uses three parameters to describe the RT distribution: mu (μ = mean of normal distribution), sigma (σ = SD of normal distribution), and tau (τ = mean and SD of the exponential tail). In one highly replicated example, children with ADHD show significant increases in tau relative to controls, which indicates a greater percentage of slow RTs^41–43^. This pattern of results may reflect more frequent attentional lapses in ADHD.

The present study takes a novel approach to attentional bias variability in PTSD by examining its relationship to more general sustained attention and top-down cognitive control processes. ABV and ICV are expected to be strongly correlated and increased in the PTSD patients, suggesting that ABV can be considered a more general variant of RT variability. This perspective is especially relevant given the known drawbacks of the ABV index^31^. The distributional measures of variability were estimated by fitting the ex-Gaussian function to the RT data. An increase in tau would indicate a greater number of unusually slow responses in the patients, perhaps reflecting more frequent off-task incidents. A correlation between tau and scores on the Cognitive Failures Questionnaire^44^ would support this interpretation. An increase in sigma would suggest greater overall variability of performance, which might be more in line with the dynamic view of fluctuating bias^37^. We also investigated whether blocks with trauma-specific negative words would differ from blocks with non-specific negative words. If the trauma-related indices show a greater enhancement in combat Veterans with PTSD, this would suggest these stimuli disrupted visual-spatial attention to a greater extent.

## Methods

### Participants

The participants were 31 Iraq and Afghanistan combat Veterans with a clinical diagnosis of PTSD, and 29 age-matched control Veterans. The data from three participants with PTSD were excluded for poor performance (see below). One control participant was excluded from the analysis when a past mTBI was revealed. Thus, the reported results include 28 in the PTSD group (26 male) and 28 in the control group (25 male). PTSD diagnosis was based on the Clinician-Administered PTSD Scale (CAPS) or semi-structured clinical interview using DSM-5 criteria^2^ and in all cases was due to combat exposure. Common mental health comorbidities (e.g., depression, generalized anxiety) were allowed. A history of mild TBI was also allowed, because many PTSD patients in this population also have mTBI(s) due to blast exposure^45^. Mild TBI was diagnosed based on standard criteria: loss of consciousness (LOC) ≤ 30 min or altered mental status; post-traumatic amnesia < 24 hrs^46^. Among the PTSD patients, 15 had experienced one or more incidents of probable mTBI and 13 reported no mTBI history.

The exclusion criteria included severe cognitive dysfunction or dementia; history of neurodevelopmental abnormalities; severe psychiatric problems (schizophrenia, bipolar disorder, schizoaffective disorder); ongoing illicit drug or alcohol abuse; history of other (non-TBI) neurological disorders; current medical illnesses that may alter mental status or disrupt participation in the study; central motor or visual deficits. The exclusion criteria above also applied to control participants. Other exclusionary conditions for controls included a history of TBI or PTSD, and current depression or anxiety.

One participant with PTSD was excluded because of issues with drowsiness and falling asleep during the session. Two other patients were extreme outliers in their accuracy (error rate 20% or greater for at least one condition, which is 8 SD outside the mean of the other patients), and were excluded.

The groups did not differ significantly in age [*t*(53.00) = 1.491, *p = *0.142], but controls had more years of education [*t*(46.79) = 3.056, *p = *0.004]. This is often due to the inability of many of the patients to return to school after their military service, and is typical of studies on this population of Veterans with PTSD (e.g.^47^). We include further analyses suggesting that the major performance indices were not significantly associated with years of education (Supplementary Materials). See Table 1 for details on demographic data.

**Table 1:** Demographic information and symptom severity scores for participants with PTSD and Controls.

	Controls (n = 28)	PTSD (n = 28)
Age (yrs)	38.32 (8.50)	35.14 (7.41) n.s
Education (yrs)	15.54 (2.22)	14.00 (1.47)**
Handedness	21 R, 7 L	24 R, 2 L, 2 amb
PCL-5	8.39 (7.23)	47.04 (16.67)***
• intrusion	2.14 (2.17)	11.29 (5.11)***
• avoidance	0.96 (1.53)	5.25 (2.49)***
• negative cognitions	2.32 (2.58)	14.54 (7.38)***
• increased arousal	2.96 (3.25)	15.96 (5.10)***
BDI	5.36 (3.41)	19.75 (11.16)***
CFQ	31.14 (10.58)	58.25 (15.99)***

Note: The means (standard deviations) are given for age, education, PCL-5, and BDI. n.s. = not significantly different from controls; **significantly different from controls at p < 0.01; ***significantly different from controls at p < 0.001. R = right, L = left; amb = ambidextrous; PCL-5 = PTSD checklist for DSM-5; BDI = Beck Depression Inventory; CFQ = Cognitive Failures Questionnaire.

The Institutional Review Board of the VA Northern California Health Care System approved the study protocol. All participants gave informed consent before starting the experiment. They were paid $20/hour for their participation plus transportation expenses. The research was conducted in accordance with the Declaration of Helsinki.

### Dot Probe Task

The stimuli were neutral, negative, and combat-related words taken from the set of Ashley *et al*.^3^. To examine the potential trauma-related specificity of bias, the negative words were from a Combat category (e.g. “gunfire”) or a General category (e.g. “poison”) in separate blocks. The design was closely based on the study of Wald *et al*.^21^. In each block, 38 pairs of negative and neutral words were presented above and below fixation, followed by a probe stimulus (one or two red dots) in the location of one of the words. Each trial began with a central fixation display (+++) visible for 500 ms, followed immediately by a word pair presented for 1000 ms. Then the probe appeared for 500 ms in the location previously occupied by either the neutral or the negative word in the pair, and the participant was instructed to press the “1” key for one dot, and the “2” key for two dots. The response window was 1500 ms, and RTs greater than that were not included. The next trial began 2000 ms after probe offset.

Each negative word in a pair was presented with a neutral word of the same length (3–8 letters for General, 3–10 letters for Combat). Negative and neutral conditions within a block were matched for frequency^48^, number of syllables, and word type (see^3^). The position (upper/lower), status (target/distractor), and target response (one/two) associated with each word were counterbalanced across four blocks, separately for the Combat and General stimulus sets. The 38 pairs in each set repeated across the four blocks, but in a different random order each time; there were short breaks between the blocks.

To maintain the participants’ attention on the words, they were instructed that a short and easy memory test would be presented after the experiment. The recognition test was administered on a sheet of paper in a forced choice format with 15 pairs of words: one word was a target and the other a foil. Participants were told that each row contained one word that was presented in the dots task and another word that was not. They were instructed to circle the word they remember seeing in the dots task. Ten of the rows had neutral target/foil pairs, and five rows had negative target/foil pairs (all from the General block). One patient did not complete the memory test and was omitted. The test sheet was missing from one control subject. Participants with PTSD correctly identified a mean of 11.9 out of 15 words, compared to 11.6 for controls (*p* = 0.699).

### Self-Report Questionnaires

All participants completed behavioral questionnaires after the session. The PTSD Checklist for DSM-5 (PCL-5) is a 20-item self-report measure that assesses the 20 DSM-5 symptoms of PTSD and may be used for screening, provisionally diagnosing, or monitoring symptom change of PTSD^49^. It has four clusters or subsets: intrusion, avoidance, negative alterations in cognition, and alterations in arousal. Symptoms are rated on a 0 to 4 scale. Preliminary validation work suggests that PTSD is present at a cut-point of 33 or greater on the PCL-5^49^. The Beck Depression Inventory (BDI) is one of the most commonly used self-report screens for major depressive disorder and has been validated with well-established psychometric properties^50^. The Cognitive Failures Questionnaire (CFQ) is a 25-item Likert-type scale used to assess the frequency with which people experience cognitive failures in everyday life^44^. Subjects rate how often such experiences have happened to them in the last 6 months on a scale of 0 (“Never”) to 4 (“Very often”) for items such as, “Do you find you forget appointments?” As expected, the two groups showed highly divergent scores on these questionnaires (Table 1), indicating higher levels of PTSD, depression, and cognitive failures in the patients.

### Statistical Analysis

RTs from correct trials were analyzed using repeated measures ANOVA with factors of Valence (negative, neutral), Block Type (combat, general), and Group (controls, patients). The bias index (neutral trials – negative trials) and attention bias variability (ABV) were also calculated, and analyzed using ANOVA with factors of Block Type and Group. A bias index with a positive value indicates attention directed towards negative stimuli, and vice versa for bias index with negative value. ABV is measured by dividing the experimental session into eight mini-blocks, determining the bias index of each mini-block, finding the SD of these, and dividing by RT. Welch’s *t*-tests were used when independent samples *t*-tests were appropriate, and the adjusted degrees of freedom are reported.

Another measure of overall response variability (but not bias variability) is the intra-individual coefficient of variation (ICV = SD/mean RT), which corrects for any baseline differences in RTs. Initial results suggested that RTs for negative words and neutral words were drawn from highly similar distributions that did not differ notably between the two valence types in either the Combat or the General blocks (see Table 2). This impression was supported by comparing the RT distributions for negative and neutral trials by fitting ex-Gaussian functions for negative and neutral trials separately. Therefore, RTs were collapsed across Valence for ICV, and analyzed using ANOVA with factors of Block Type and Group.

**Table 2:** Behavioral Performance in the Dot Probe Task. Means of individual subjects’ reaction times (top) and error rates (bottom) are shown for Controls and participants with PTSD. Trauma-related negative words (Combat) and non-trauma negative words (General) were presented in separate blocks, along with the appropriately matched set of neutral words.

**RT**	Combat		General	
**(ms)**	negative	neutral	negative	neutral
Controls	514.1 (61.7)	514.3 (59.4)	511.2 (61.1)	511.9 (60.7)
PTSD	589.7 (148.0)	589.9 (141.4)	572.4 (131.5)	577.6 (136.3)
**Errors**	**Combat**		**General**	
**(%)**	**negative**	**neutral**	**negative**	**neutral**
Controls	1.88 (1.77)	1.46 (1.91)	1.38 (1.25)	1.18 (1.54)
PTSD	2.45 (2.88)	1.52 (2.30)	1.28 (1.70)	1.70 (1.69)

Note: The means (standard deviations) are in milliseconds for RT (reaction time) and in percentages for error rate.

The distributional measures of central tendency and variability were estimated by fitting the ex-Gaussian function to the RT data. The ex-Gaussian parameters mu, sigma, and tau were calculated using the QMPE program^51^ and entered into separate ANOVAs with factors of Block Type and Group. Spearman correlations were used to examine the relationship between scores on the self-reported symptom instruments and the variability indices, with α set at 0.005. We also calculated Bayes factors for the major results using JASP statistical software version 0.8.1.1 (see Supplementary Material).

### Data Availability

The datasets generated during and/or analyzed during the current study are not publicly available due to institutional privacy policies but are available from the corresponding author on reasonable request.

## Results

### Reaction Time

Participants with PTSD were slower overall than the control participants [F(1,54) = 6.354, *p* = 0.015, η_p_^2^ = 0.105]. However, neither the patients nor the controls showed an attentional bias towards *or* away from negative words in either the Combat blocks or General blocks (Table 2): the effects of Valence [F(1,54) = 1.669, *p* = 0.202, η_p_^2^ = 0.030] and Valence × Block Type [F(1,54) = 0.551, *p* = 0.461, η_p_^2^ = 0.010] were not significant. Furthermore, neither of these variables interacted with Group: [F(1,54) = 0.763, *p* = 0.386, η_p_^2^ = 0.014] and [F(1,54) = 0.355, *p* = 0.554, η_p_^2^ = 0.007], respectively. The patients showed more variability in RT, however, at both the group level (Table 2) and the individual level. Within-subject SD was greater in the PTSD participants than in control participants [F(1,54) = 9.490, *p* = 0.003, η_p_^2^ = 0.149], but Group did not interact with Valence [F(1,54) = 0.078, *p* = 0.782, η_p_^2^ = 0.001] or Block Type [F(1,54) = 0.074, *p* = 0.787, η_p_^2^ = 0.001].

**Figure 1:**
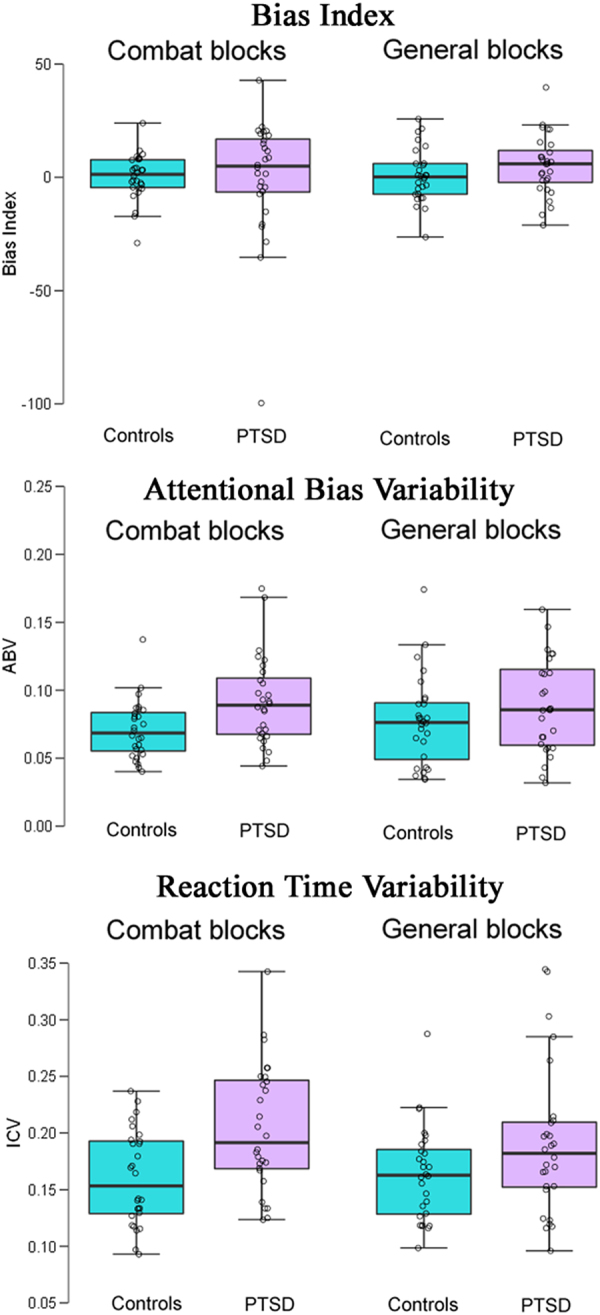
Box plots for the main measures in the study. Top: Bias Index = mean RT for neutral words – mean RT for negative words. Middle: Attention Bias Variability (ABV) = SD of 8 bias mini-blocks/mean RT. Bottom: Intra-individual Coefficient of Variation (ICV) = mean RT/mean SD. Controls are shown in the cyan bars, PTSD in pale violet.

Figure 1 (top) illustrates the bias index (neutral RT – negative RT). Welch’s *t*-tests for independent samples revealed no significant group differences for either the Combat blocks [*t*(35.03) = 0.014, *p* = 0.989, *d* = 0.004] or the General blocks [*t*(53.08) = 1.293, *p* = 0.202, *d* = 0.345].

### Response Variability

**Figure 2:**
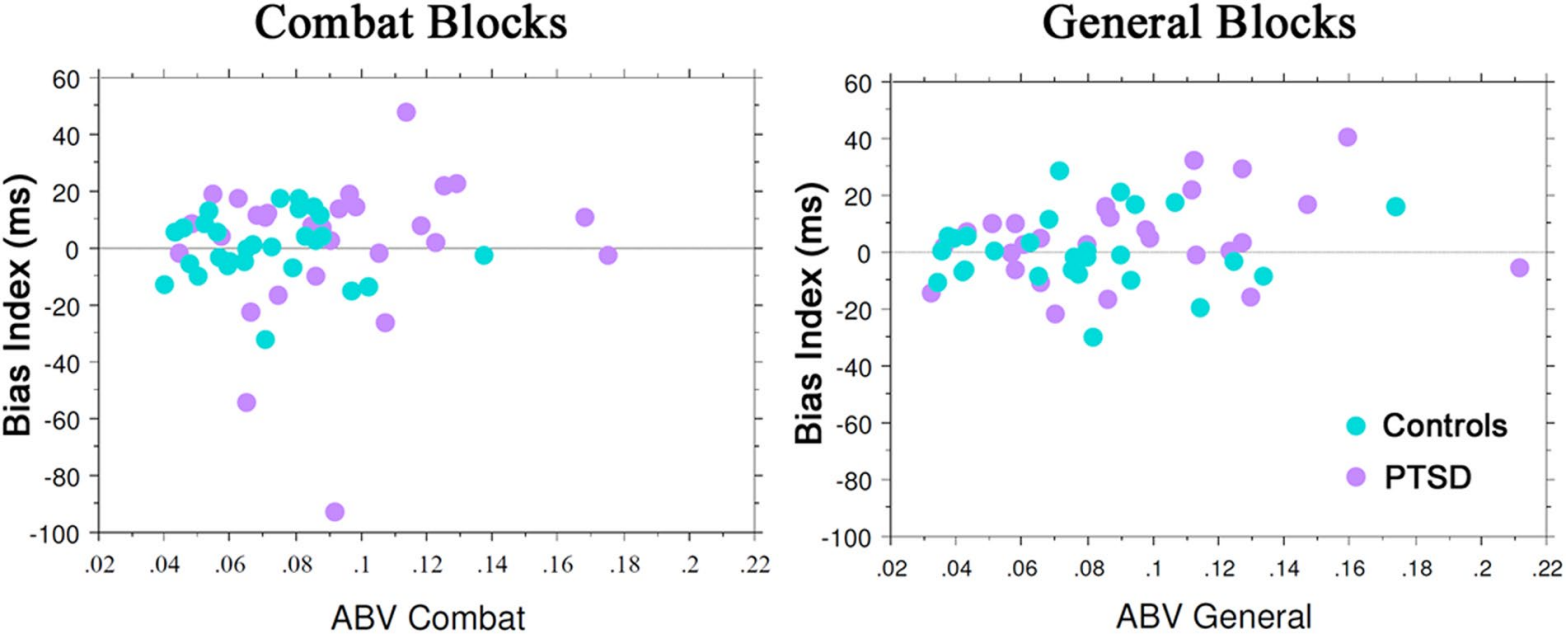
Scatterplots showing the correlation between the Bias Index (in milliseconds) vs. Attention Bias Variability (ABV) for the Combat blocks (left) and the General blocks (right). Controls are depicted by the cyan dots, PTSD by pale violet. Correlations are not reported, because bias can take on both positive and negative values.

Results for the ABV index suggested that participants with PTSD showed greater fluctuations in attentional bias than controls in the Combat blocks *and* the General blocks (Fig. 1, middle). This observation was supported by a significant main effect of Group [F(1,54) = 6.659, *p* = 0.013, η_p_^2^ = 0.110], but no further interaction with Block Type [F(1,54) = 0.341, *p* = 0.562, η_p_^2^ = 0.006]. However, if the overall difference between RTs to negative and neutral words is close to zero (Fig. 1, top), any fluctuation from zero may be more reflective of other sources of variance than of true variability in bias^31^. Figure 2 illustrates this point by plotting the relationship between ABV and the bias index. A given participant with a bias index near zero can show bias variability at either the low end or the high end of the distribution.

As indicated by the within-subject SD results above, PTSD patients showed an overall increase in RT variability, and this effect was not based on apparent fluctuations in bias. The ICV index (Fig. 1, bottom) corrects for differences in mean RT. Within each Block Type, responses to negative and neutral stimuli were drawn from the same RT distribution, so we collapsed across Valence. The ICV analysis revealed a main effect of Group [F(1,54) = 7.586, *p* = 0.008, η_p_^2^ = 0.123] and no significant interaction with Block Type [F(1,54) = 2.352, *p* = 0.131, η_p_^2^ = 0.042]. Notably, the two indices of variability were highly correlated with each other: ABV and ICV for the Combat blocks (*rho* = 0.690, *p* < 0.001) and the General blocks (*rho* = 0.852, *p* < 0.001).

To examine the shape of the RT distribution, the three ex-Gaussian parameters were entered into separate ANOVAs with factors of Block Type and Group. Mu was numerically larger in the patients (Table 3), indicating slower RTs for the normal component of the curve, but this was not significant [F(1,54) = 3.638, *p* = 0.062, η_p_^2^ = 0.063]. Sigma was significantly larger in the patients, reflecting greater SD for the normal component [F(1,54) = 7.656, *p* = 0.008, η_p_^2^ = 0.124]. Likewise, tau was significantly larger in the patients [F(1,54) = 8.251, *p* = 0.006, η_p_^2^ = 0.133]. Taken together, these results suggest that increased response variability in PTSD was not entirely due to a preponderance of long RTs in the rightward tail, but also included more variability around the Gaussian distribution. The Group × Block Type interactions were not significant for any of the measures (p’s > 0.4), suggesting similar patterns for the Combat and General blocks. These findings were supported by Bayesian repeated measures ANOVAs (Supplementary Material). Finally, there was overwhelming evidence in favor of a correlation between tau and the ABV index (Table S1, Supplementary Material).

**Table 3:** The ex-Gaussian parameters for the Combat blocks and the General blocks in controls and participants with PTSD.

Condition	Controls Mean (SD)	PTSD Mean (SD)
Combat mu	440.6 (43.7)	477.7 (95.0)
General mu	441.4 (45.2)	476.9 (97.2)
Combat sigma	41.1 (13.0)	58.8 (30.2)**
General sigma	43.7 (16.2)	57.1 (31.0)*
Combat tau	71.2 (30.4)	112.6 (73.5)**
General tau	67.9 (28.8)	98.4 (62.5)*

Note: The means (standard deviations) are in milliseconds. The parameters are mu (μ = mean of normal distribution), sigma (σ = SD of normal distribution), and tau (τ = mean and SD of the exponential tail). *p ≤ 0.05; **p < 0.01 (Welch’s t-tests for independent samples).

### Errors

Error rates were low (Table 2) and did not differ significantly between the two groups [F(1,54) = 0.549, *p* = 0.462, η_p_^2^ = 0.010], nor did Group interact with Valence [F(1,54) = 0.023, *p* = 0.881, η_p_^2^ = 0.000] or Block Type [F(1,54) = 0.039, *p* = 0.844, η_p_^2^ = 0.001]. A modest Valence × Block Type interaction was observed [F(1,54) = 4.413, *p* = 0.040, η_p_^2^ = 0.076], due to higher errors for probes replacing negative combat words relative to other stimuli. This result suggests a minor disruption in performance when the probe appears in the former location of a combat word, but this interpretation is speculative and should be made cautiously in light of floor effects for errors.

### Correlation with Symptom Ratings

Because the self-reported symptoms of PTSD and depression were so highly correlated (*rho* = 0.855, *p* < 0.001), we only report results for the PCL-5. The bias index was not significantly associated with PTSD symptom severity for either Block Type: Combat (*rho* = 0.200, *p* = 0.139); General (*rho* = 0.200, *p* = 0.138). (Note, however, that the bias index can have positive or negative values). ABV in the Combat blocks was correlated with PCL-5 scores (*rho* = 0.449, *p* < 0.001), but ABV in the General blocks was not (*rho* = 0.175, *p* = 0.198). Likewise, ICV in the Combat blocks was correlated with PCL-5 scores (*rho* = 0.456, *p* < 0.001), but this was not the case for the General blocks (*rho* = 0.235, *p* = 0.081). These findings could suggest that the variability indices in blocks with trauma-related words show a specific relationship with PTSD symptoms, but this requires that the correlations are significantly different from one another. Using the method of^52^, the difference between two dependent correlations with one variable in common^53^ was determined, revealing that in neither case did the Combat vs. General comparison reach significance: ABV (*z* = 1.748, *p* = 0.080) and ICV (*z* = 1.938, *p* = 0.053).

Correlations between CFQ scores and ICV would indicate that everyday failures in perception, memory, and motor function are associated with more variable performance in the dot probe. ICV in the Combat blocks was correlated with CFQ scores (*rho* = 0.465, *p* < 0.001), but this did not reach significance for the General blocks (*rho* = 0.342, *p* = 0.010), nor for ABV in the Combat (*rho* = 0.343, *p* = 0.010) or General blocks (*rho* = 0.238, *p* = 0.077). More specifically, correlations between CFQ and ex-Gaussian tau would be consistent with the notion that unusually slow responses reflect attentional lapses during the task. Tau in the Combat blocks was in fact correlated with CFQ scores (*rho* = 0.438, *p* < 0.001), but not significantly for the General blocks (*rho* = 0.335, *p* = 0.012). Cognitive failures were not related to sigma in the Combat (*rho* = 0.302, *p* = 0.024) or General blocks (*rho* = 0.200, *p* = 0.139). We revisited these correlations using Bayesian analyses (Table S2, Supplementary Material), which corroborated the results described here.

## Discussion

Participants with PTSD did not show an attentional bias either towards or away from negative words in a dot probe task, replicating an increasing number of studies^19,11,30^. Moreover, this lack of bias was true for negative words from a general category, *and* for specific trauma words related to combat exposure. Conversely, attention bias variability was enhanced in participants with PTSD compared to controls. However, the absence of bias in both groups, along with slower RTs and larger SDs in the patients, renders this group difference problematic^31^. The intra-individual coefficient of variation, a bias-neutral index of response variability, was also elevated in the patients. The ex-Gaussian parameters sigma and tau were increased in the PTSD group as well, indicating that variability in the normal RT distribution and the exponential component (rightward tail) were both affected. These results question whether behavioral variability in the dot probe task is due to a vacillation in “bias” towards and away from threatening stimuli, rather than an overall inconsistency in responding.

The lack of attentional bias stands in contrast to our prior study using these same words in the emotional Stroop task^3^, although the design of that task does not allow one to disentangle an increased bias towards threat from difficulties with disengaging from threat. The present results provide additional evidence that the traditional threat index from the dot probe task is not a suitable measure of attentional bias in PTSD. Although some researchers have provided suggestions for improving the measure^54^, previous studies have demonstrated the unreliability of the bias index in anxiety disorders^29^ and control populations^27,28^. For these reasons, some investigators have moved away from the bias index and instead prefer the new measures of bias variability.

The ABV index was developed to assess within-session fluctuations in attention bias^19^, and this metric does differentiate between PTSD and control groups in the dot probe task^19,11,30,55^ a finding we replicated in the present experiment. However, if the mean bias index is not significantly different from zero (i.e., no bias), any deviation from zero can be considered “noise” or variability due to other sources. RT in this task is a reflection of where the participant is attending on a given trial, but it also includes the elements of stimulus discrimination (one or two dots), decision, and response to the probe. While the dots are simple stimuli they still require a response, around which are sources of variability that go beyond biased spatial attention.

Importantly, ABV is sensitive to increases in SD in the absence of attentional bias^31^, which was indeed the case for the patients relative to the controls. RT data simulations that introduced relatively small group differences in SD yielded overly high percentages of significant *t*-tests when mean RT was kept constant^31^. More broadly, ABV can be problematic even when attentional bias is present. This is not surprising, given the formula for ABV (SD of bias across 8 bins/mean RT). The participants with PTSD also had greater mean RTs than controls (an increase in the denominator would *reduce* the value of ABV), but the simulations suggest that RT likely had less of an impact on ABV than the significant increase in SD^31^. Furthermore, two crucial pieces of information are lost when calculating ABV: the sign or direction of bias (towards/away from threat) and the magnitude of bias^31^. If the source of the increased variance indicated by the ABV index is uncertain, are other measures of response variability more informative?

ICV was increased in the combat Veterans with PTSD, confirming that their overall performance on the task was less consistent than controls. This replicates the results from a letter GNG task, an inhibitory task with neutral stimuli^38^. That experiment demonstrated that response variability was increased in this PTSD population in the absence of threatening or trauma-related stimuli, suggesting a more general impairment in top-down control processes that maintain steady performance. The relationship between bias variability scores in the present study and overall SD was illustrated by the correlation between ABV (a bias-based measure) and ICV (an SD-based measure). However, the dot probe task differs from the GNG in that there is a visual-spatial component, a choice RT on each trial (tapping response selection processes), and no requirement to inhibit responses to infrequent stimuli.

In some ways, proponents’ interpretations of the ABV and TL-BS indices may seem like a mere semantic distinction from the position put forth here. An overall increase in RT variability can also be viewed as a fluctuation of attention between negative and neutral stimuli. One weakness of the present study is that there were no separate blocks with neutral-neutral trials only. That condition could help discriminate between threat-related bias variability and a more general increase in RT variability in the absence of threat. Some investigators have tried to consider response variability on neutral-neutral trials intermixed with neutral-negative trials, but used mean RT rather than SD^37,56^. A better approach would be to compare ICV for the two trial types when presented in separate blocks, so “carry-over” effects^57^ from threat trials do not impact neutral-only trials.

The ex-Gaussian parameters sigma and tau were both elevated in the patients, which reveals that excessively slow RTs can account for some (but not all) of the increase in response variability. A similar pattern was observed in participants with adult ADHD^58^, unlike the tau-specific effect seen in childhood ADHD. The increase in tau may reflect more frequent attentional lapses, consistent with the idea that participants with PTSD had more occasions of waning focus on the task. A number of studies have quantified tau to examine the role of attentional lapses in aging, mood disorders, substance dependence, and childhood ADHD^34,59–61^. The literature on sigma is less well-developed, but an increase in this parameter reflects greater variability in performance around the normal RT distribution. This could be consistent with an account of attentional bias that fluctuates towards and away from threat, but the ABV index in its current formulation cannot detect this possibility. Furthermore, the Bayesian analyses provided overwhelming evidence that ABV is strongly correlated with tau, which supports its relation to more general difficulties with sustained attention processes. However, other researchers have cautioned against a neat mapping of ex-Gaussian parameters onto cognitive processes^62^, so the current interpretation remains tentative.

The variability measures were related to the severity of clinical symptoms. ABV and ICV were both positively correlated with PCL-5 scores, but the bias index was not. Other investigators have reported correlations between ABV and PTSD symptoms in studies that used trauma words, general threat words, and angry faces as negative stimuli^19,30^ suggesting that ABV is capturing some aspect of performance associated with illness severity. Since ICV reflects this relationship as well, we conclude that fluctuations in attentional bias cannot be the sole driver of this effect. Instead, we suggest a relationship between the amount of personal distress and a decline in executive control which contributes to less efficient cognitive function. This view is consistent with the “mental noise” hypothesis, in which negative affective states serve as a distraction from stable task performance^35^.

PTSD symptoms were not uniquely correlated with unusually slow responses, however, since higher scores on the PCL-5 were associated with larger values of both sigma and tau (especially in the Combat blocks). On the other hand, there was strong support for a relationship between CFQ scores and tau, suggesting that everyday cognitive failures were associated with more frequent instances of waning focus during the task. In an earlier study, McVay and Kane^63^ used thought-sampling procedures to demonstrate that attentional lapses contribute to tau. Mind wandering was measured by periodically asking participants if they were experiencing task-unrelated thoughts. These subjective attentional lapses were correlated with tau, but not with sigma^63^.

There was little evidence that the observed increases in variability were specific to blocks containing trauma-related words, because these indices were also elevated in blocks with generically negative words. This is in accord with prior studies that have used trauma-specific and non-specific stimuli^19,11,30^. The lack of trauma-specificity provides support for a disruption in more generic attention processes^12^. A recent study of deployed soldiers found that TL-BS measures of bias variability were predictive of post-traumatic stress symptoms as a function of the number of intervening traumatic experiences, but the effects were not specific to threatening stimuli^64^. TL-BS scores for happy faces and angry faces showed similar predictive values in the absence of bias^64^, suggesting a potential relationship to overall response variability. In the present study, many of the correlations between symptom severity and variability were larger in blocks that contained Combat stimuli, but the discrepancies were not great enough to decisively conclude that the relationships were trauma-specific.

This study had several limitations. Comorbidities such as mTBI and depression may complicate interpretation of the results. To address the former, analyses comparing PTSD patients with and without mTBI did not reveal significant differences (see Supplementary Materials), although the number of participants in each group was low. On the other hand, it wasn’t possible to separate the effects of PTSD symptoms from those of depression, because scores on the PCL-5 and BDI were too highly correlated to disentangle. Another limitation is that the participants with PTSD had fewer years of education than controls, which may have influenced the results. However, the major dependent measures were not correlated with years of education, indicating the latter could not account for the group differences. Another shortcoming is that the present results may be specific to PTSD and not applicable to other populations with anxiety disorders. Finally, results in combat Veterans may not generlize to PTSD in civilian populations.

## Conclusions

Participants with PTSD did not show an attentional bias either towards or away from negative words in a dot probe task, a finding which was unexpected when we initiated the study. In the intervening time, several papers reporting this null effect had appeared in the literature (e.g.^11^). The other new advance in the field was the development of indices to measure the dynamic nature of attentional bias, which was no longer considered a fixed or static phenomenon^37^. Here we replicated earlier findings of enhanced attentional bias variability in PTSD patients, and its correlation with symptom severity^19,30,55^. However, it is unclear whether ABV is actually measuring variability in bias, a concern that was highlighted by RT simulation studies^31^. Our current results demonstrate an overall increase in response variability in PTSD that is not specific to threat-related biases in spatial attention. Instead, we suggest a deficit in the top-down cognitive control processes needed to maintain consistent performance, which might contribute to the persistence of PTSD symptoms^65^.

## Electronic supplementary material

Supplementary Materials
