# Supplementary material for: Enhanced Attentional Bias Variability in Post-Traumatic Stress Disorder and its Relationship to More General Impairments in Cognitive Control
Source: Sci Rep. 2017 Nov 6;7:14559. doi: 10.1038/s41598-017-15226-7 (PMC5673957; doi:10.1038/s41598-017-15226-7)
Supplement: Supplementary file 1 — Supplementary Materials [file 41598_2017_15226_MOESM1_ESM.pdf]

Enhanced Attentional Bias Variability in Post-Traumatic Stress Disorder and its Relationship to More General Impairments in Cognitive Control

Diane Swick & Victoria Ashley

Supplementary Results

Effects of mTBI

Mild TBI is a very common co-morbidity in OEF/OIF combat Veterans with PTSD (Carlson et al., 2011). In the current study, 15 of the PTSD patients reported having mTBI(s), while 13 PTSD patients did not report any mTBI. To determine whether PTSD participants with mTBI performed worse than those without mTBI, we conducted 2 x 2 ANOVAs with factors of Group and Block Type. There were no significant main effects for any of the measures: Bias Index [$F(1,26)= 0.012$, $p=0.912$, $\eta_p^2 =.000$], ABV [$F(1,26)= 0.065$, $p=0.801$, $\eta_p^2 =.002$], or ICV [$F(1,26)= 0.553$, $p=0.464$, $\eta_p^2 =.021$]. There was a modest Group × Block Type interaction for ABV [$F(1,26)= 5.133$, $p=0.032$, $\eta_p^2 =.165$], but in light of the low numbers of subjects in each group we will not discuss this further.

Effects of Education

The controls had more years of education than the patients, so we wished to determine whether this discrepancy affected the results. Having a diagnosis of PTSD could be casually related to lower educational attainment in some cases. For this reason (and others outlined by Miller & Chapman, 2001), ANCOVA including education as a covariate is inappropriate. Instead, we ran Spearman correlations to assess the potential relationship between the dependent variables and years of education. The results suggested no significant correlations between education and task performance. For the General blocks, the correlations were *rho*=.022, *p*=0.873 for the bias index, *rho*=-0.170, *p*=0.210 for ABV, and *rho*=-0.166, *p*=.221 for ICV. For the Combat blocks, the correlations were *rho*=-0.095, *p*=0.486 for the bias index, *rho*=-0.195,

p=0.150 for ABV, and *rho*=-0.334, *p*=0.012 for ICV (which did not meet the p<.005 corrected level of significance).

Bayesian Analyses

We quantified the strength of evidence for the variability measures using Bayesian hypothesis tests. Bayes Factors (BF_{10}) were calculated using JASP statistical software version 0.8.1.1 (JASP Team, 2017). $BF_{10} < 1$ provides evidence in favor of the null hypothesis (H_0), while $BF_{10} > 1$ favors the alternate hypothesis (H_1). First, we ran JZS Bayes Factor repeated measures ANOVAs (Morey & Rouder, 2015; Rouder et al. 2012) with default prior scales. For ABV, the main effects model with Group only ($BF_{10} = 3.542$) was preferred to the Block × Group interaction model ($BF_{10} = 0.251$). This means that the Group difference in ABV was 3.542 times more likely than $H_{0.}$ Overall, this can be considered "mild" evidence (Wagenmakers et al., 2017) in favor of the Group model, although this finding is bolstered by the fact that it replicates previous results.

For ICV, the main effects model with Group ($BF_{10} = 6.159$) was 6.159 times more likely than H_0, providing "moderate" support for $H_{1.}$ Evidence in favor of the interaction model (BF_{10} = 1.356) was ambiguous or "anecdotal", but the main effects model was still preferred (6.159/1.356 = 4.542). For both sigma ($BF_{10} = 6.033$) and tau ($BF_{10} = 7.764$), the data provided "moderate" support for a main effect of Group over H_0, with less evidence for the interaction models, with $BF_{10} = 0.433$ and 1.291, respectively.

Does attentional bias variability show an especially strong relationship with either sigma or tau? Bayesian correlations (Kendall's Tau-B) between ABV and the ex-Gaussian variability measures are reported in Table S1, separately for Combat and General blocks. There was, in fact, overwhelming support for correlations between ABV and tau, suggesting that the former reflects

especially slow responses in the rightward tail of the RT distribution.

We also wished to examine the relationships between the experimental measures and clinical symptoms. Bayesian correlations (Kendall's Tau-B) between self-reported symptom ratings and the variability measures are reported in Table S2, separately for Combat and General blocks. These results support the main findings using Spearman correlations. There was strong evidence favoring associations between PTSD symptoms and ABV, ICV, sigma, and tau for the Combat blocks. The evidence supporting correlations between CFQ and variability in the Combat block were very strong (ICV), strong (tau), and mild (ABV). For the General block, there was mild support for associations between CFQ and ICV, as well as tau.

Supplementary References

Carlson, K. F., *et al*. Prevalence, assessment, and treatment of mild traumatic brain injury and posttraumatic stress disorder: A systematic review of the evidence. *J Head Trauma Rehab* **26**, 103-115 (2011).

JASP Team. JASP (Version 0.8.1.1) [Computer software] (2017).

Miller, G. A., & Chapman, J. P. Misunderstanding analysis of covariance. *J. Abnorm. Psych.* **110**, 40 (2001).

Morey, R. D., & Rouder, J. N. *BayesFactor 0.9.11-1*. Comprehensive R Archive Network. (2015).

Rouder, J. N., Morey, R. D., Speckman, P. L., & Province, J. M. Default Bayes factors for ANOVA designs. *J. Math. Psychol.* **56**, 356-374 (2012).

Wagenmakers, E. J., *et al*. Bayesian inference for psychology. part II: Example applications with JASP. *Psychon Bull Rev* 1-19 [published online: 06 July 2017].

Table S1. Bayes Factors (BF_{10}) for Bayesian correlations (Kendall's Tau-B) between Attentional Bias Variability (ABV) and the ex-Gaussian measures of variability in the dot probe task.

	**BF_{10}**	
ABV Combat – Combat sigma	3.253	*
ABV Combat – Combat tau	1090.569	****
ABV General – General sigma	19.579	**
ABV General – General tau	1.924e +8	****

$BF_{10} > 1$ favors H_1 and < 1 favors H_0. * Mild, ** Strong, and **** Extreme evidence in favor of H_1 (Wagenmakers et al., 2017). For example, evidence in favor of a correlation between ABV and tau in the Combat blocks was 1090.569 times more likely than $H_{0.}$

Table S2. Bayes Factors (BF_{10}) for Bayesian correlations (Kendall's Tau-B) between self-reported symptom ratings and experimental measures of variability in the dot probe task.

	**Combat ABV**	**Combat ICV**	**General ABV**	**General ICV**
PCL-5	47.728 ***	51.451 ***	0.475	0.721
CFQ	4.121 *	95.772 ***	0.935	5.223 *

	**Combat sigma**	**Combat tau**	**General sigma**	**General tau**
PCL-5	14.256 **	17.636 **	1.592	1.592
CFQ	1.807	28.409 **	0.430	4.426 *

$BF_{10} > 1$ favors H_1 and < 1 favors H_0. * Mild, ** Strong, and *** Very Strong evidence in favor of H_1 (Wagenmakers et al., 2017). PCL-5 = post-traumatic stress disorder checklist for DSM-5; CFQ = Cognitive Failures Questionnaire; ABV = attentional bias variability; ICV = intra-individual coefficient of variation.
